# Ergostane-type steroids from mushrooms of *Pleurotus* genus

**DOI:** 10.1007/s11418-024-01872-5

**Published:** 2025-01-17

**Authors:** Takashi Kikuchi

**Affiliations:** Faculty of Pharmaceutical Sciences, Toho University, Miyama 2-2-1, Funabashi, Chiba 274-8510 Japan

**Keywords:** *Pleurotus*, Ergostane, Steroid, Seco, *Abeo*, Biological activity

## Abstract

Steroids are physiologically important compounds for animals, plants, and fungi, and they have significantly contributed to drug discovery for many years. Fungi mainly biosynthesize ergostane-type steroids such as ergosterol. However, after the basic skeleton is biosynthesized, chemical transformations can lead to the cleavage or rearrangement of the fundamental skeleton of steroids. The cleaved (seco) and rearranged (*abeo*) steroids are chemically and pharmacologically intriguing because they often exhibit biological activities. As ergostane-type steroids including the normal, seco, and *abeo* types have been isolated from the mushrooms of the *Pleurotus* genus, this review focuses on them as a resource of ergostane-type steroids, providing their distribution, structure determination, and biological activity.

**Graphical abstract:**

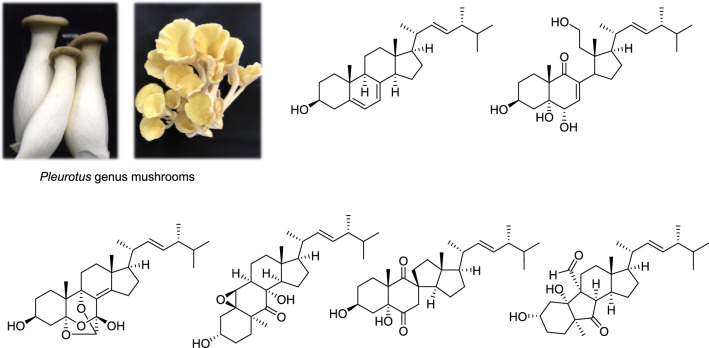

## Introduction

Ergostane-type steroids are a class of naturally occurring steroids derived from ergosterol (**1**), a principal sterol in fungi [1]. These compounds exhibit significant chemical diversity, resulting not only from the general oxidative and reductive modifications of the ergostane skeleton but also from carbon skeletal cleavage and rearrangement [2]. Moreover, ergostane-type steroids have reportedly exhibited various biological activities including anticancer, antioxidant, anti-inflammatory, and antimicrobial activities [3].

Mushrooms of the *Pleurotus* genus (Pleurotaceae) are among the most widely cultivated and consumed edible fungi globally; they are one of the major commercially important mushrooms supplied worldwide, along with *Lentinula*, *Auricularia*, *Agaricus*, and *Flammulina*. Moreover, in recent years, production in Japan has significantly increased [4]. This genus encompasses several species, including *Pleurotus ostreatus*, *P. eryngii*, and *P. pulmonarius*, which are renowned for their culinary and medicinal properties [5]. The chemical constituents of *Pleurotus* genus mushrooms include polysaccharides, phenolic compounds, amino acid derivatives, and ergostane-type steroids [5].

Despite the value of *Pleurotus* genus mushrooms as resources for ergostane-type steroids with diverse structures, there has been no comprehensive review regarding them. This review aims to summarize recent research progress on ergostane-type steroids from the *Pleurotus* genus mushrooms, highlighting their chemical diversity, isolation and structure determination, and biological activities.

## Chemical structures

Ergostane-type steroids are ubiquitously present in *Pleurotus* genus mushrooms; however, only a limited number of species have been chemically investigated in detail (Table 1). Despite this, ergostane-type steroids exhibiting diverse chemical structures have been discovered. In this review, these steroids are classified into three types based on their structures: those maintaining the basic ergostane skeleton (normal ergostane-type), those resulting from skeletal cleavage (secoergostane-type), and those forming new skeletons through rearrangement reactions are termed (*abeo*-ergostane-type).

**Table 1:** Distribution of ergostane-type steroids in *Pleurotus* genus mushroom

Species	Normal ergostane	Secoergostane	*Abeo*-ergostane	Literatures
*P. cornucopiae* var. *citrinopileatus*	**✓**	**✓**	**✓**	[6, 7]
*P. cystidiosus*	**✓**			[8, 9]
*P. djamor*	**✓**			[10]
*P. eous*	**✓**			[9]
*P. eryngii*	**✓**	**✓**	**✓**	[11–17]
*P. ostreatus*	**✓**			[18–23]
*P. sajor-caju*	**✓**			[24, 25]
*P. tuber-regium*	**✓**			[26]

**Fig. 1:**
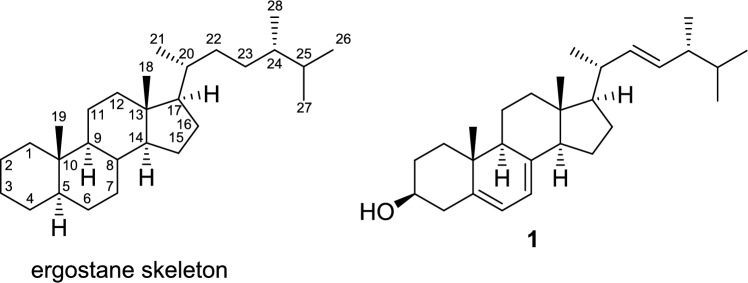
Ergostane skeleton and ergosterol (**1**)

**Fig. 2:**
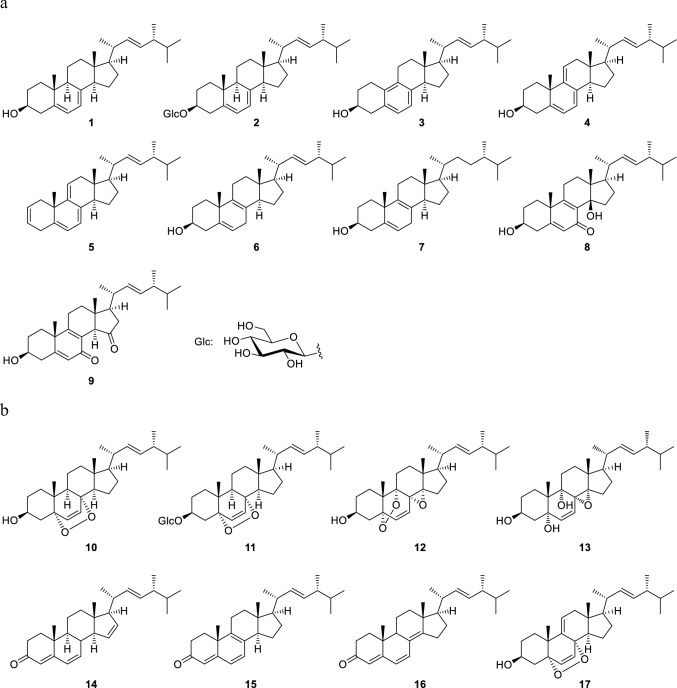

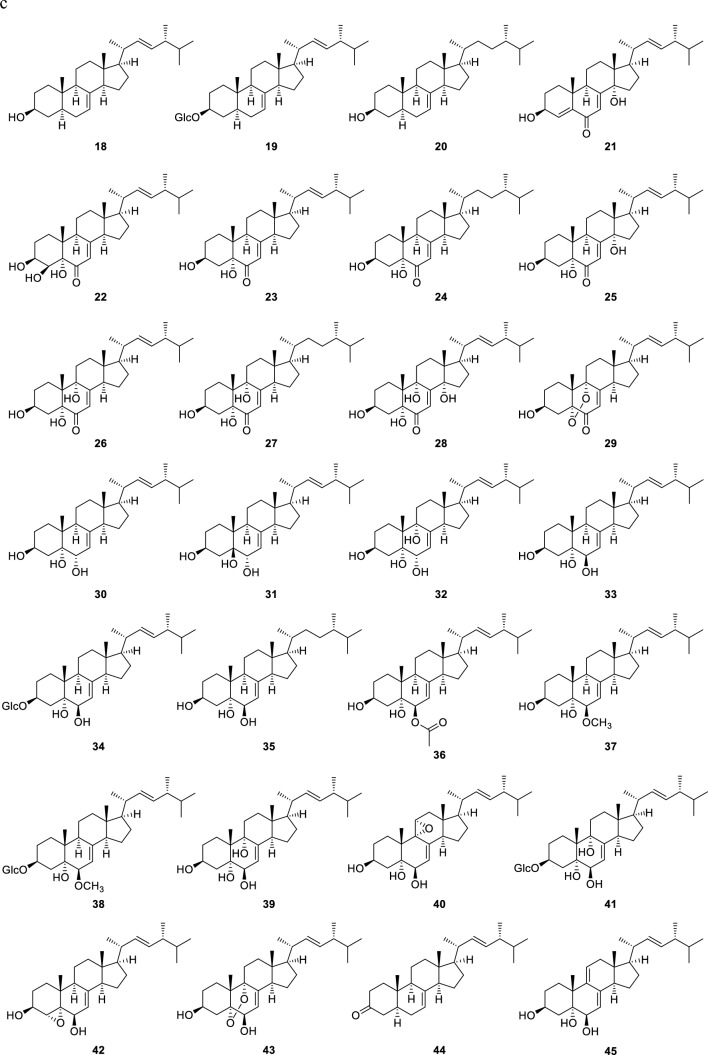

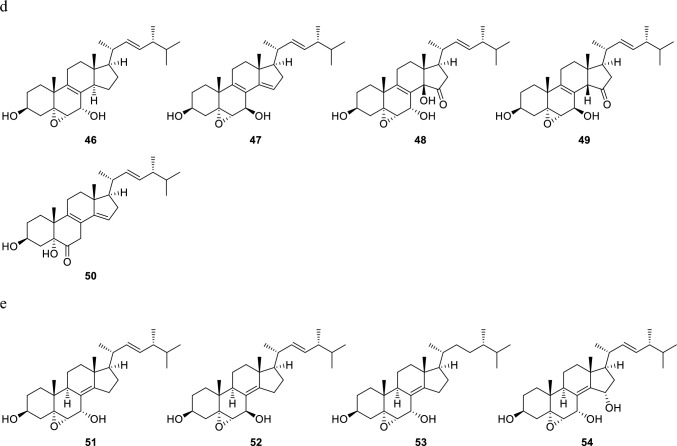
Normal ergostane-type steroids from *Pleurotus* genus mushrooms. **a** Steroids with double bonds at C-5 and C-7; C-5, C-7, and C-9; C-5, C-7, and C-9 (11); and C-5 and C-8; **b** steroids with a double bond or double bonds at C-6; C-6 and C-8; C-6 and C-8 (14); and C-6 and C-9 (11); **c** steroids with a double bond or double bonds at C-7 and C-7 and C-9 (11); **d** steroids with a double bond at C-8; **e** steroids with a double bond at C-8 (14)

Ergostane-type steroids possess a cyclopentanoperhydrophenanthrene carbon skeleton formed by A/B/C/D rings, with side chains of C-20–C-28 positions. Most of ergostane-type steroids from *Pleurotus* genus mushrooms have transfused A/B and C/D rings, with double bonds at C-22 of the side chain and between the C-7 and C-8, C-8 and C-9, or C-8 and C-14 positions (Fig. 1). The structures of the normal ergostane-type steroid compounds are made up of functional groups through hydroxylation, epoxidation, olefination, etc., at positions C-5–C-9 of the B ring. They are shown in Fig. 2 according to the location of the double bonds in the B and C rings.

The most typical normal-type compound, ergosterol (**1**), has double bonds at the C-5 and C-7 positions in the B ring. There is also a glucoside of ergosterol at the C-3 position (**2**). In addition, there are compounds with double bonds at the C-5 and C-8(9) positions in the B ring exist (**6**, **7**), compounds with conjugated triene structures (**4**, **5**), conjugated 5,8-dien-7-one structures (**8**, **9**), and a compound lacking a methyl group at the C-19 position that possessing the B aromatic ring (**3**) (Fig. 2a). Concerning ergostanes with a double bond at the C-6 position, compounds with a 5,8-peroxy structure (**10**, **11**, **17**), a 5,9-peroxy structure (**12**), an 8,14-epoxy group (**12**, **13**), and a conjugated structure due to a carbonyl group at the C-3 position and double bonds at C-4 and C-6 positions (**14** − **16**) have been reported (Fig. 2b). Ergostanes with a double bond at the C-7 position exhibit diverse structures (**18** − **45**) (Fig. 2c). Most of these compounds have a hydroxy group at the C-5α position and either a carbonyl or a hydroxy group at the C-6 position, with some exceptions, such as those with a 5,9-peroxy structure (**29**, **43**), a 5β-OH (**31**), or a 4,5-epoxy structure (**42**). In addition, some compounds have a hydroxy group at the C-4β (**22**) or C-9α positions (**26** − **28**, **32**, **39**, and **41**), or a methoxy (**37**, **38**) or an acetoxy group (**36**) at the C-6β position. There are also compounds with functional groups such as a 3-*O*-glucoside (**19**, **34**, **38**, **41**) and a 4-ene structure (**21**) in the A ring, and a 9,11-ene structure (**45**), a 9,11-epoxy group (**40**), and a 14-hydroxy group (**21**, **25**, **28**) in the C ring. Ergostanes with a double bond between C-8 and C-9 positions (**46** − **49**) have been reported to possess oxygen functional groups such as hydroxy, and epoxy or carbonyl groups at the C-5, C-6, and C-7 positions (Fig. 2d). In addition, compounds (**47** − **50**) have 14-ene, or 14-OH and 15-one structures in the D ring. Of these compounds (**48**, **49**) have *cis*-fused C/D rings due to the presence of β-H or β-OH at the C-14 position, whereas compounds with a double bond between C-8 and C-14 positions can have oxygen functional groups such as 5,6-epoxy and 7-hydroxy groups (**51** − **54**)(Fig. 2e).

**Fig. 3:**
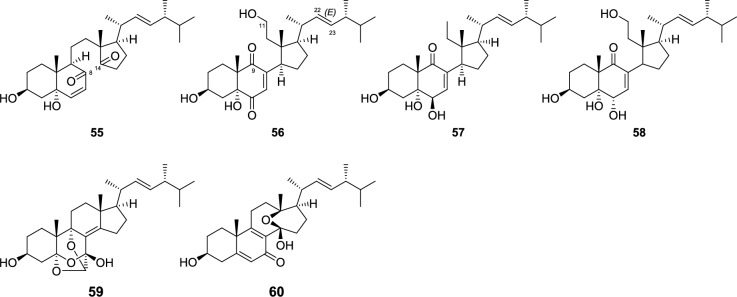
Seco ergostane-type steroids from *Pleurotus* genus mushrooms

The secoergostane-type steroids (**55**–**60**) isolated from the *Pleurotus* genus mushrooms include 5,6-seco, 13,14-seco, 8,14-seco, and 9,11-seco-ergostanes, and their sources are *P. eryngii* and/or *P. cornucopiae* var. *citrinopileatus* (Fig. 3). In the 9,11-secoergostanes (**56**–**58**), there is a carbonyl group at the C-9 position, whereas in the 8,14-secoergostane (**55**), there are carbonyl groups at the C-8 and C-14 positions, and they have a C ring opening structure. Conversely, in that of eringiacetal A (**59**), an oxygen atom is inserted between C-5 and C-6, and in that of eringiacetal B (**60**), a 13,14-seco compound, an oxygen atom is inserted between C-13 and C-14.

**Fig. 4:**
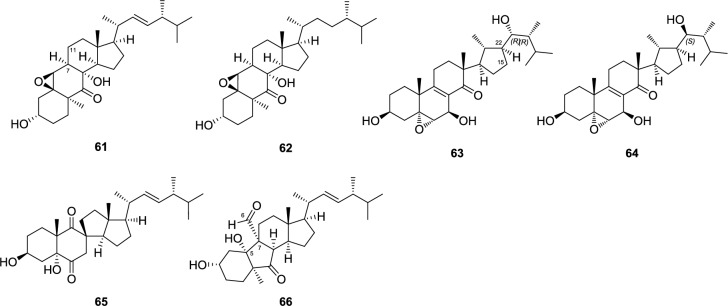
Structures of *abeo*-ergostanes from *Pleurotus* genus mushrooms

The *abeo*-ergostane-types, such as an 11(9 → 7)*abeo*-ergostane steroid, 15(14 → 22)*abeo*-ergostanes, an 11(9 → 8)*abeo*-ergostane and a 5(6 → 7), 11(9 → 7)di*abeo*-ergostane, are isolated from *P. eryngii* or *P. cornucopiae* var. *citrinopileatus* (Fig. 4). The 11(9 → 7) *abeo*-ergostane steroids, such as pleurocins A (**61**) and B (**62**), have a structure in which the A/B rings are flipped vertically, with the C-9 of the ergostane skeleton bonded to C-7 instead of C-11. A carbonyl group is present at the C-9 position. The 15(14 → 22) *abeo*-ergostanes (**63**, **64**) of C-15 bond to C-22 on the side chain instead of C-14, forming a five-membered ring. A carbonyl group is present at the C-15 position. Pleurocorol A (**65**), an 11(9 → 8) *abeo*-ergostane, has a structure where a six-membered B ring and a five-membered C ring are connected at the spiro carbon at the C-8 position. Regarding pleurocorol B (**66**) of the 5(6 → 7), 11(9 → 7) di*abeo*-ergostane, C-5 bonds to C-7 instead of C-6, and additionally, the carbon at C-11 bonds to C-7 instead of C-9, resulting in a vertical flip of the A/B ring and a five-membered B ring structure. A carbonyl group is present at the C-9 position.

## Extraction and isolation

To isolate ergostane-type steroids from *Pleurotus* genus, the mushrooms were extracted with methanol, 80–95% ethanol in water, or acetone [6, 8, 9, 12–17, 20, 25, 26]. Thereafter, the extract is then partitioned with ethyl acetate and water, or chloroform and water. Silica gel column chromatography is employed to separate the ethyl acetate fraction or the chloroform fraction. Hexane, dichloromethane, diethyl ether or ethyl acetate extractions were also performed, followed by silica gel or neutral alumina column chromatography of the extract [8–11, 18, 19, 21–23]. Solvents such as acetone, chloroform dichloromethane, ethanol, ethyl acetate, hexane, methanol, petrol, petroleum ether or toluene including their combined solvent systems of chloroform–ethyl acetate chloroform–methanol, dichloromethane–acetone, dichloromethane–methanol, hexane–benzene, hexane–dichloromethane, hexane–ethyl acetate, hexane–acetone, petrol–acetone, petroleum ether–ethyl acetate, or toluene–ethyl acetate are used for column chromatography. Ergostane-type steroids are isolated from the obtained fractions using high-performance liquid chromatography (HPLC) with octadecylsilyl columns (eluent: methanol–water or acetonitrile–water) or HPLC with silica gel columns (eluent: hexane–ethyl acetate). Preparative thin-layer chromatography [9, 21] or the Chromatotron [8] is also used for purification. For the recrystallization of compounds, solvents such as acetone, chloroform–methanol, petroleum–acetone, or petroleum–ethyl acetate are used.

## Structural determination

The chemical structure of ergostane-type steroids is typically determined by nuclear magnetic resonance (NMR) spectral analysis. The ergostane-type steroids from the *Pleurotus* genus mushrooms typically show two singlets of tertiary methyl groups at the C-18 and C-19 positions. Further, they exhibit four doublets of secondary methyl groups at C-21, C-26, C-27, and C-28, and two doublets of doublets of the double bond between C-22 and C-23. Except for a hydroxy or epoxy group at the C-4 position, the ergostane-type steroids of the *Pleurotus* genus mushrooms have a hydroxy group at the C-3 position of the A ring, and the hydroxymethine at the C-3 position appears as a triplet of triplets in ^1^H NMR spectra [6, 15–17]. This splitting pattern is also observed in steroids derived from sources other than *Pleurotus* genus mushrooms [27–29].

**Fig. 5:**
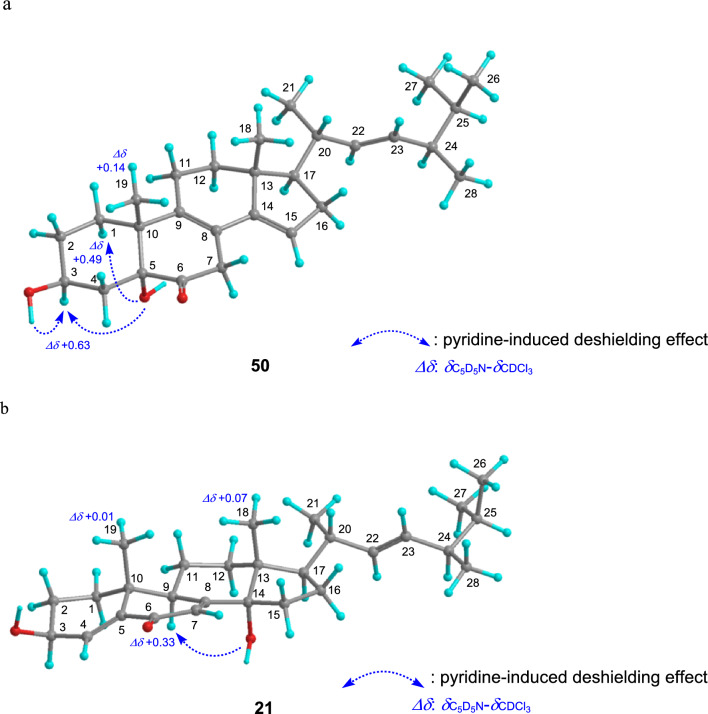
The pyridine-inducing deshielding effects in compounds **50** (a) and **21** (b)

A tertiary hydroxyl group is sometimes present at the C-5 position. This configuration can be determined by confirming the pyridine-induced deshielding effect on surrounding protons such as H-1α, H-3, and H-9. That is, near one hydroxyl group, the δ_H_ shifts approximately 0.3 ppm downfield in C_5_D_5_N measurements compared with that in CDCl_3_ or CD_3_OD measurements (Fig. 5a) [30–35]. Further, this can be applied to the determination of 14-OH orientation such as **21** (Fig. 5b).

In case an epoxy group exists at C-5, the *δ*_C_ of C-5 under CDCl_3_ measurement is typically observed at approximately 63–68 ppm [6, 15, 18], whereas when it is a hydroxyl group, signals above *δ*_C_ 70 ppm are observed [6, 8, 17, 18, 20]. Further, several compounds with chemical modifications such as olefination or epoxidation between C-8 and C-14 or C-9 and C-11 and hydroxylation or carbonylation at positions C-14 and C-15 of the C and D rings, have been isolated. The configuration at C-20 in ergostanes isolated from the *Pleurotus* genus mushrooms has been consistently identified as *S* configuration. This can be determined using NOESY, showing NOE correlations such as H-16α/H-22, H_3_-18/H-20, and H_3_-28/H-14 based on the calculated stable conformations of (20*R*) and (20*S*) [6]. However, the configuration at C-24 in ergost-22,23-ene structures can be determined from ^13^C NMR signals, as there are differences in the chemical shift values of *δ*_C_, particularly in the CDCl_3_ of 24*R* [*δ*_C_ 42.9 (C-24) and 17.7 (C-28)] and 24*S* [*δ*_C_ 43.2 (C-24) and 18.1 (C-28)] methylcholestane-type steroids [36].

**Fig. 6:**
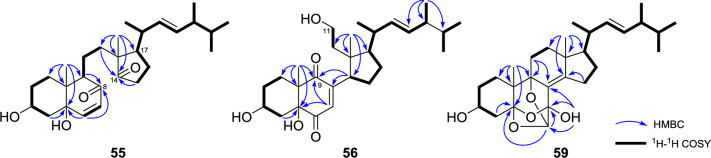
Key HMBC and ^1^H–.^1^H COSY correlations of secoergostanes (**55–59**)

For 8,14-seco and 9,11-secoergostanes, such as (3β,5α,22*E*)-3,5-dihydroxy-8,14-secoergosta-6,22-diene-8,14-dione (**55**), (3β,5α,22*E*)-3,5,11-trihydroxy-9,11-secoergosta-7,22-diene-6,9-dione (**56**) [6], (3β,5α,6β,22*E*)-3,5,6-trihydroxy-9,11-secoergosta-7,22-dien-9-one (**57**) [16], and (3β,5α,6α,22*E*)-3,5,6,11-tetrahydroxy-9,11-secoergosta-7,22-dien-9-one (**58**) [17], correlations from the methyl groups of H_3_-18 or H_3_-19 to the keto carbonyl group are observed in the HMBC spectrum. This facilitates the prediction of C–C bond cleavage with the keto carbonyl group (Fig. 6). In addition, for 9,11-secoergostanes, it is noteworthy that the CH_2_ signals at C-11 are replaced by oxymethylene or primary methyl group signals. For the configuration of side chains, the method of determining those of the normal ergost-22-ene side chain can be applied. In NMR spectrum analysis, when overlapping signals hinder analysis, changing the NMR measurement solvent can be an effective means to deduce the correct structure. For the structure determination of (3β,5α,22*E*)-3,5,11-trihydroxy-9,11-secoergosta-7,22-diene-6,9-dione (**56**), the signals of H-22 and H-23 on the side chain overlap in CDCl_3_ measurement; however, in C_5_D_5_N measurement, it shows* J* = 15.6 Hz, and it has been determined to have an *E* configuration [6].

Conversely, in 5,6-seco and 13,14-secoergostanes, the C–C bonds between C-5 and C-6, and C-13 and C-14 are cleaved, with ether bridges existing between them. Eringiacetal A (**59**), a 5,6-secoergostane, possesses a cage-like structure with three acetal carbons forming three ether bridges, which is unique among other steroids [13]. In the structural analysis of this compound, the HMBCs from of H-6 to the acetal carbons of C-5 and C-7, from 7-OH to the acetal carbons of C-6 and C-7, and the olefinic carbon of C-8 are observed. These HMBCs were crucial in elucidating the cage-like structure of the B ring of the steroid skeleton (Fig. 6).

**Fig. 7:**
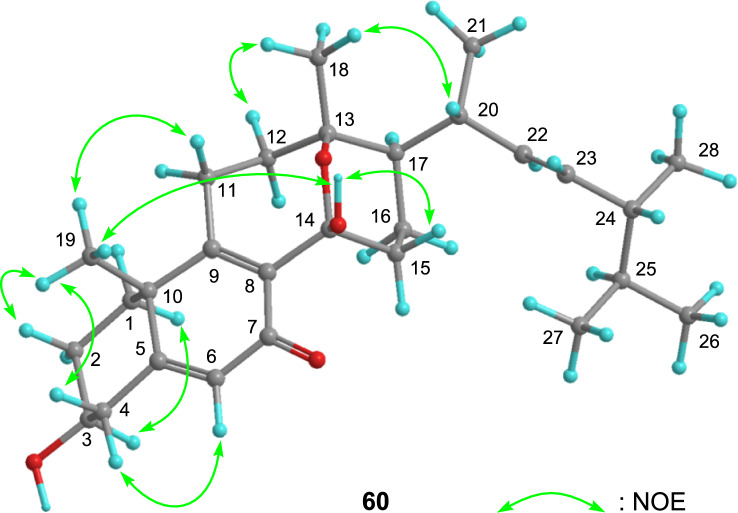
Key NOE correlations of eringiacetal B (**60**)

In addition to HMBC, NOESY has proven to be an important tool in the structure determination of 13,14-secoergostane, eringiacetal B (**60**) [14]. Correlations were observed from H_3_-18 to a tertiary oxycarbon (C-13) and an acetal carbon (C-14), suggesting the presence of C–C bond cleavage and ether bond formation. Regarding the conformation at the C-13 and C-14 positions, the configuration of the C/D ring in this compound cannot be (13*R*,14*S*) or (13*S*,14*R*) because of the strain, which means that the hydroxy group at the C-14 position is too hindered to be stable [37, 38]. It is proposed that the *cis* configuration, i.e., CH_3_-18–C-13–O–C-14–OH, takes a W-shaped conformation. The relative configuration was determined by combining NOE correlations (Fig. 7).

In the structural elucidation of the *abeo*-steroids isolated from the *Pleurotus* genus mushrooms, the HMBCs from H_3_-18 or H_3_-19 methyl to carbonyl groups are observed as in the seco type, suggesting the cleavage of the ergostane skeleton. However, ^1^H–^1^H COSY and HMBCs confirm the formation of new C–C bonds. In the ^1^H and ^13^C NMR spectra of pleurocin A (**61**), an 11(9 → 7)*abeo*-ergostane steroid, four tertiary methyl groups and two secondary methyl groups are observed, like in normal ergostane-type steroids [14]. Conversely, the HMBC from H_3_-19 to the carbonyl group at C-9 and the ^1^H–^1^H COSYs of H-6–H-7–H_2_-11 have been observed. These correlations indicate the inversion of the A/B rings and the presence of a C–C bond between C-7 and C-11. Simultaneously, pleurocin B (**62**), which is the 22,23-dihydro derivative, has been isolated and structurally determined [14].

In addition, the 15(14 → 22)*abeo*-ergostanes, strophasterols E (**63**) and F (**64**) [16], have been isolated from *P. eryngii* mushrooms. In the HMBC experiments of the 15(14 → 22)*abeo*-ergostanes, correlations from H_3_-18 to the carbonyl group at C-14 are observed. Furthermore, ^1^H–^1^H COSYs between H-15–H-22(–H-20)–H-23 have been observed, revealing the cleavage of the C–C bond between C-14 and C-15 and the formation of the cyclopentane ring by the bonding of C-15 and C-22. Strophasterols E (**63**) and F (**64**) are the 23*R* and 23*S* epimers.

Regarding pleurocorol A (**65**) having a six-membered B ring and a five-membered C ring connected by a spiro carbon at the C-8 position, the HMBCs from H_2_-7, H_2_-11, H-12α, and H-14 to quaternary carbon of C-8 have been observed. This indicated that the C–C bond exists between C-11 and C-8 instead of C-11 and C-9 [7]. Conversely, in pleurocorol B (**66**) with a five-membered B ring and a six-membered C ring, HMBCs from the proton of the formyl group, H-6, to C-7 and C-8, and from H-8 and H-11β to the formyl carbon, C-6, were observed. This indicated the presence of a formyl group at C-6 and the cleavage of the C–C bond between C-5 and C-6 [7].

**Fig. 8:**
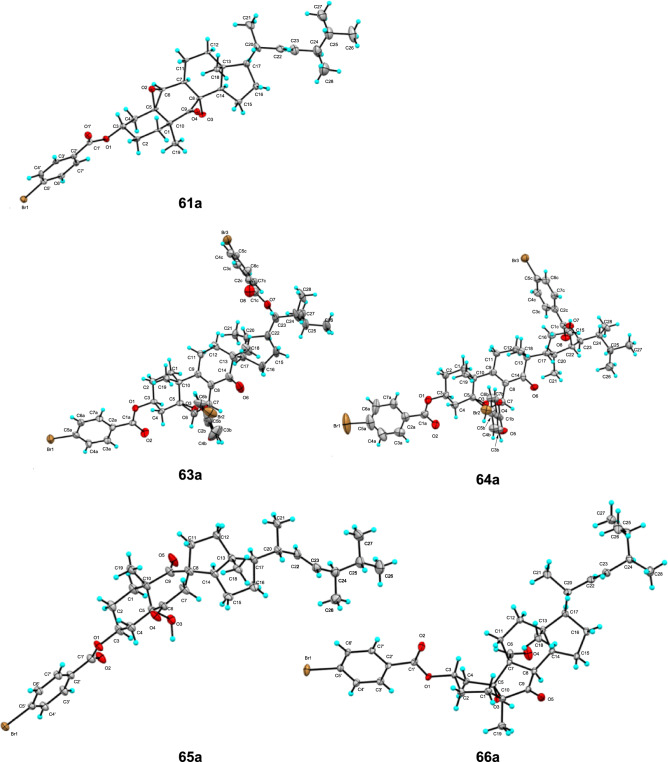
Oak-Ridge thermal ellipsoid plots of compounds **61a** and **63a**–**66a**

X-ray crystallography, along with mass spectrometry and NMR, is an effective means for determining detailed structures, and also enables the determination of absolute configuration, although it requires suitable crystals. If the crystallization of *abeo*-ergostane steroids is difficult, the preparation of its *p*-bromobenzoate derivative is an effective method for crystallization. The absolute configurations of pleurocin A (**61**), strophasterols E (**63**) and F (**64**), and pleurocorols A (**65**) and B (**66**) have been confirmed through the X-ray crystallographic analysis of their *p*-bromobenzoate derivative (**61a**, **63a**, **64a**, **65a**, and **66a**) (Fig. 8) [7, 14, 16].

## Biosynthesis

Ergostane-type steroids are biosynthesized from lanosterol, which is generated from squalene [39]. Through alkylation at the C-24 position, demethylation at the C-14 position, demethylation at the C-4 position, and structural modifications of the side chain and B ring, ergosterol (**1**), a representative ergostane-type steroid, is synthesized. The biosynthesis of *abeo*-type steroids has not yet been elucidated; however, it is presumed as follows.

**Fig. 9:**
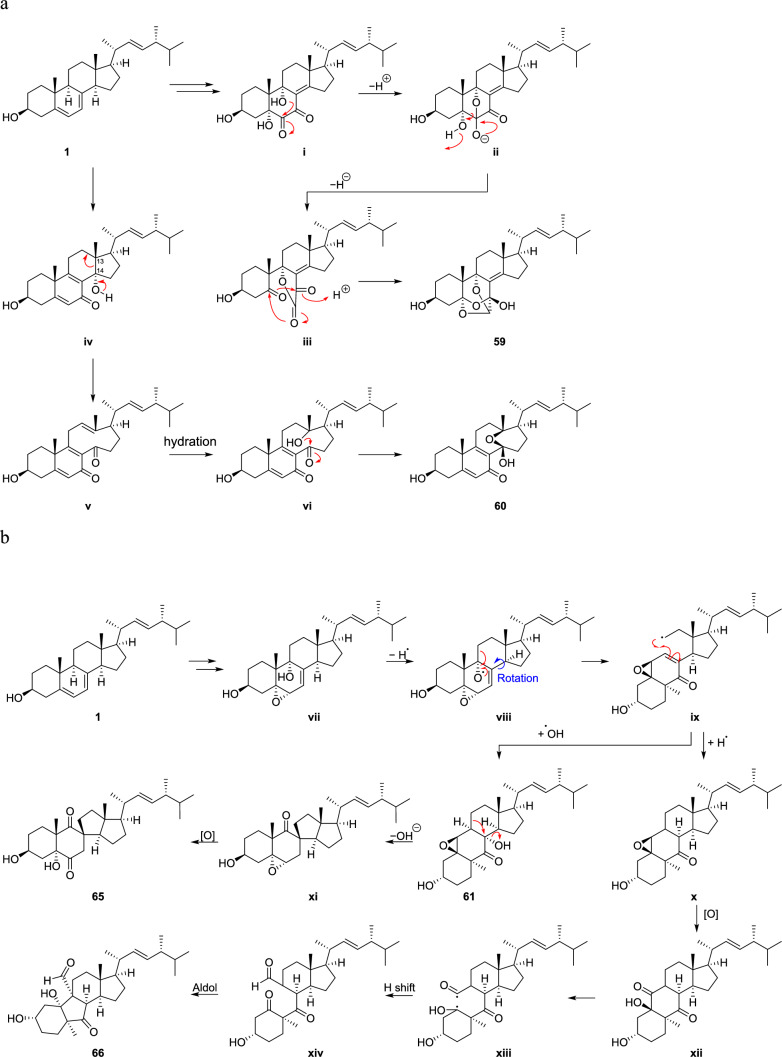
Possible biosynthetic pathway of *abeo*-ergostane steroids **59** and **60** (a) and **65** and **66** (b)

The biosynthetic pathways of eringiacetals A (**59**) and B (**60**) are both predicted to be derived from ergosterol (**1**) (Fig. 9a) [13, 14]. Intermediate **i**, synthesized from ergosterol (**1**), undergoes acetalization at the C-6 position with 9-OH, resulting in **ii**. Subsequently, the cleavage of the C–C bond between C-5 and C-6 affords **iii**. It is proposed that eringiacetal A (**59**) is further synthesized through acetalization at the C-5 and C-7 positions. Regarding eringiacetal B (**60**), it is hypothesized that during the conversion of the hydroxyl group of **iv** to a carbonyl group, the C–C bond at positions 13 and 14 undergoes cleavage, and affords **v**. Thereafter, eringiacetal B (**60**) is synthesized through water addition and acetalization at the C-14 position.

It is considered that pleurocorols A (**65**) and B (**66**) are generated from pleurocin A (**61**) and **x**, formed through radical formation from ergosterol (**1**) [7]. The dehydroxylation of pleurocin A (**61**), followed by translocation and oxidation, produces pleurocorol A (**65**). Conversely, the oxidation of **x** results in the cleavage of the C-5–C-6 bond, producing diradical **xiii**. Furthermore, it is presumed that pleurocorol B (**66**) is generated through hydrogen translocation and aldol reaction (Fig. 9b).

## Chemical synthesis

The chemical synthesis of natural compounds is important for proving the structure of natural compounds and for supplying them for further biological activity evaluation and pharmacological experiments. Regarding the steroids of the *Pleurotus* genus mushrooms, the *abeo*-ergostane type has been the target of synthetic research, and pleurocins A (**61**) and B (**62**) and strophasterols E (**63**) and F (**64**) have been synthesized.

The synthesis of pleurocins A (**61**) and B (**62**) has been achieved by Heinze and Heretsch through oxidative radical cyclization [40], whereas Sato and Kuwahara et al. have synthesized strophasterol E (**63**) and F (**64**) [41, 42]. This synthesis involves a 1,3-dipolar cycloaddition reaction with a nitrile oxide intermediate, selectively and diastereoselectively introducing a cyclopentane ring and a C-23 oxygen functional group. In addition, a position-selective and diastereoselective selenohydroxylation of the olefin intermediate under thermal conditions is performed. In addition to strophasterols E (**63**) and F (**64**), they have synthesized the proposed structure of glaucoposterol A isolated from *Cortinarius glaucopus* and proposed that glaucoposterol A is strophasterol F (**64**).

## Biological activity

Although there have been few reports on the biological activity of steroids from *Pleurotus* genus mushrooms, it has been reported that they inhibit nitric oxide (NO) production.

Macrophages may serve as a potential therapeutic target for inflammatory diseases [43]. Activated macrophages produce pro-inflammatory agents such as NO, reactive oxygen species, interleukin-1 beta, tumor necrosis factor-alpha, and other inflammatory compounds, which are crucial for biological defense. Nonetheless, the overexpression of these agents is linked to diseases such as osteoarthritis, rheumatoid arthritis, and diabetes, as their heightened production can trigger severe or chronic inflammation [43]. Normal ergostane-type (**48**–**50**, **52**) [6, 16, 17], secoergostane-type (**56**, **58**, **60**), and *abeo*-ergostane-type (**61**, **62**, **65**, **66**) steroids exhibit inhibitory effects on NO production without cytotoxicity at tested concentrations. Among them, normal ergostane-type compounds (**48** and **49**) have a double bond at the C-8 position, a β-H or β-OH group at the C-14, and a carbonyl group at the C-15 position. Further, 9,11-secoergostane-type compounds (**56** and **58**) have a carbonyl group at the C-9 position and a hydroxyl group at the C-11 position. However, the structure–activity relationships have not been fully elucidated.

Other biological activities have been reported, including aromatase inhibitory activity and cytotoxicity against cancer cells. Ten normal ergostane-type steroids have been evaluated for their aromatase inhibitory activities. Of these, ergosterol (**1**) and brazzein (**37**) exhibit comparable inhibitory activities to those of aminoglutethimide. In addition, compounds (**9**, **14**, **18**, and **47**) have shown moderate activities [15]. The cytotoxic activity of *abeo*-steroids (**65**, **66**) against erythromyelogenous leukemic cells K562 and the corresponding P-glycoprotein overexpression cells K562/ADR has been evaluated. Pleurocorol B (**66**) exhibits moderate activities against both cell lines [7].

## Conclusion

This review focused on ergostane-type steroids including normal, seco, and *abeo* types from *Pleurotus* genus mushrooms. Fungi, through the biosynthetic pathways of their secondary metabolites, offer promising natural resources with novel carbon skeletons. Although the content of such compounds in natural resources is typically low, in recent years, significant efforts have been made toward the total synthesis of some seco and *abeo* steroids using effective strategies [44]. These studies are very useful for providing a stable supply, as well as for proving their structures and elucidating their biosynthetic pathways. Seco and *abeo* steroids have the potential for biological activities different from conventional cyclopentanoperhydrophenanthrene skeleton steroids, and novel mechanisms of the biological activities can be anticipated because of their unique structures. These naturally occurring bioactive compounds with novel structures can be used for drug discovery research.
